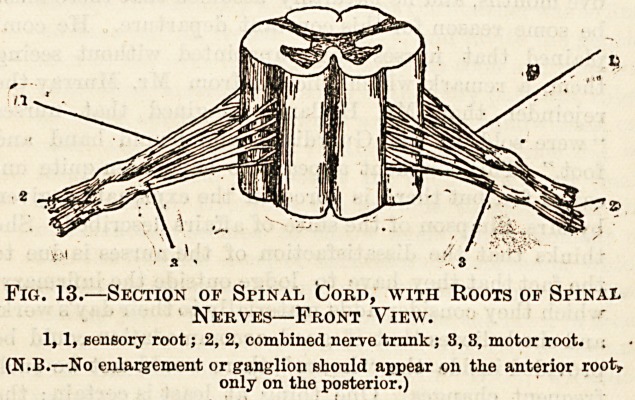# "The Hospital" Nursing Mirror

**Published:** 1900-05-26

**Authors:** 


"The Hospital" Nursing Mirror.

Being the Nursing Section of "The Hospital."

[Contributions for this Section of "The Hospital" should be addressed to the Editor, The Hospital, 28 & 29, Southampton Street, Strand, London, W.C., and should have the word "Nursing" plainly written in left-hand top corner of the envelope.]

### Notes on News from the Nursing World.

### THE HOWARD DE WALDEN HOME.

On Saturday afternoon the Princess Louise, Duchess of Argyll, paid a private visit to the Howard de Walden Nurses' Home and Club, of which she is the patron. The Princess was received in the entrance-hall by the nurses, Miss Wells (the matron of the home) and Miss Gethen (secretary to the Nurses' Co-operation), and a bouquet of red roses and lilies was presented by one of the senior nurses on behalf of the staff. Her Royal Highness made a thorough inspection of the building, including the spacious kitchens, and expressed satisfaction with the arrangements for the comfort of everyone, as well as approval of the furniture, fittings, and scheme of decoration. After taking tea in the restaurant, the Princess made an appropriate and graceful speech to the assembled nurses, who cheered her enthusiastically. On the previous Thursday the nurses and their friends were invited to inspect their new possession, and many availed themselves of the opportunity. Amongst the guests were Miss Gordon, of St. Thomas's; Miss H. A. C. Gordon, of Charing Cross; Miss Hughes, Miss de Pledge, Miss C. J. Wood, Miss Paul, and many others. Very warm admiration was generally expressed at all the arrangements.

### NURSING AT THE GABLES HOSPITAL.

When Mr. and Mrs. Alfred Cooper opened The Gables Hospital at Surbiton for the reception of the wounded soldiers returning from the front on the "Princess of Wales" hospital ship it was thought that the services of a matron would suffice. But, as The Gables is now used as a relief hospital for Netley and very serious cases are received, it has been found necessary to have the constant attendance of three nurses. A fair idea of the nature of their duties may be gathered from the fact that on Saturday four cases were operated upon in the new operating theatre. One was that of Private Cowell, who had a bullet through his head at Spion Kop, and while lying wounded was shot through the left thigh by a Mauser bullet, being further injured by the bursting of a shell. Another case was that of Corporal McHugh, who was wounded from a shell in the right orbit at Hussa Hill; a third that of Corporal Cox, who received a shell wound in his left leg at Potgieters Drift; and a fourth that of Sergeant Garland, who received a Mauser bullet in his right foot at Globlers Kloof. Frequent operations give the increased nursing staff plenty to do, and Dr. Beville, the house surgeon, is in constant attendance.

### A NOTE FROM BLOEMFONTEIN.

A Sister of the Army Reserve Nursing Service, who has just arrived at the General Hospital, Bloemfontein, writes:—

"Those who have given hints to nurses about to proceed to the war do not seem to have laid sufficient stress upon the advantage of taking old underclothing for board ship, which can be thrown away when soiled. As it is usually only sixteen days to the Cape, no large supply is needful, but for East London enough for an extra week must be arranged. For use in travelling up country, nurses who are provided with lunch baskets, tea, tins of condensed milk, spiritine, and a tin of biscuits, also tinned meats, find themselves exceedingly popular at meal times! It is quite impossible to procure food just when you most need it sometimes, therefore it is advisable to have something to fall back upon. As to drugs, by all means take some phenacetin and caffeine tabloids, and above all some diarrhœa mixture and a bottle of brandy—not necessarily a big bottle. I brought no stimulants with me of any kind, and often regretted the absence of it. Money should be in gold, not in £5 notes."

Having described the voyage, which she found very pleasant, only marred by the death of three of the soldiers from pneumonia, whom the nurses were not allowed to take care of, though they longed to do so, the same correspondent says:—

"Our journey from Cape Town here was a long one—700 to 800 miles—taking us from nine p.m. on Wednesday to one a.m. on Sunday. Though the journey is interesting it is somewhat of a trial, owing to the small and close accommodation, the limited supply of water and food, and the dust and heat. On our arrival we were most kindly entertained to breakfast by Canon Orford, his mother, and sister. For the following day and a half we were allowed to be idle, but after that our duty was portioned out. I had a large ward of enteric patients to nurse, others had surgical cases, either English or Boer, to look after; but one and all were glad to get to work again in earnest."

### NURSING AT MARITZBURG.

Writing to us from the Base Hospital, Maritzburg, an Army Reserve nursing sister dwells on the delights of "the open-air sort of life." She says: "I cannot go from one patient to another without being outside; I cannot fetch anything without going to another building. It is all out in the open, and I do not know, I am sure, how I shall ever again be able to live inside the four walls of a ward." On the other hand, our correspondent misses the hospital bath, and observes that nurses at home are not half thankful enough for their hospital bath-rooms, with plenty of water, hot and cold, and plenty of room. "Still," she continues, "even ablutionary difficulties do not damp my ardour. Nor yet the ants. The latter inundate us; they march their armies right into the middle of our food, and have pitched battles before our eyes. Many of my typhoid patients are getting better. I have two surgical patients as well, one with a very bad arm and the other with septicæmia. Both are doing fairly well."

### DEATH OF A NURSE AT THE FRONT.

We learn, with much regret, of the death of Sister Florence Bell, of the Army Nursing Reserve, in South Africa. Miss Bell greatly rejoiced when she was selected for service at the front. She went out by the same steamer as Lord Roberts, and after a week's sojourn at the Wynberg Hospital she was despatched to Modder River. There she worked hard and happily, and her letters to her friends at home showed the same bright interest in passing events and the same care for her professional duties which distinguished her at the Great Northern Hospital, where she was trained. It was a great sorrow to many when letters recently received announced her death on April 8th at the civil hospital, Kimberley, after a week's illness from enteric fever. Miss Bell had been sent to Kimberley as soon as she fell ill, and was taken to the civil hospital as affording the best facilities for nursing. She was only 30 years of age, and her death is deeply lamented by a large circle of friends and acquaintances.

### CONCERT IN AID OF THE METROPOLITAN HOSPITAL, KINGSLAND ROAD, N.E.

A VERY successful concert was given in aid of this institution on Tuesday, May 22nd, at Queen's Hall. The programme was a very good one, and out of the great number of well-known artistes who proffered their services it is hard to single out any particular ones as being the most prominent. Suffice to say, Miss Marie Dainton surpassed herself in her clever imitations of well-known actresses; Mr. Charles Capper delighted a large and enthusiastic audience with his remarkable whistling; whilst Messrs. Hayden Coffin, R. G. Knowles, Fred Upton, and Lewis Waller amply justified the expectation of enjoyment which their names forecasted. H.R.H. the Duchess of Connaught was present, and was loudly cheered on entering the hall.

### MORE EMBARRASSING THAN THE BOERS.

A CORRESPONDENT from one of the Natal seaports writes to us: "We have just had a delightful little Ladysmith dinner, our guests being a doctor, a nurse, and a civilian, who are staying here, all lately recovered from enteric. They 'reminisced' to our hearts' content, and were most gay and bright. The nurse is still very white and puffy, and trembles dreadfully if she has to stand for any length of time, but she refuses to be condoled with about her health, and has only one complaint to make—to wit, that the gratitude of the fond parents whose sons she has nursed is overwhelming. She finds their attentions much more embarrassing, she says, than any in the shape of shot and shell which the Boers used to pay her, because they are so much more difficult to get away from."

### THE RELIEF OF MAFEKING.—AT A GENERAL HOSPITAL.

A NURSE at St. Mary's Hospital writes: "The relief of Mafeking has been celebrated here in a way which caused great rejoicing amongst the patients. All the men were supplied by the secretary with pipes and tobacco, and the women and children with bags of sweets and chocolate crêmes. The men—especially the old grey-haired 'daddies'—were brimful of joy; it was as good as a tonic to look at their faces. One patient in the casualty ward told us that both her son and brother were in Mafeking, and that when she heard the good news on Saturday night she just went out into the street and shouted with all her heart and soul. 'Nurse,' said she, 'I could do nothing but hollar to think of my dear boy. He has often been to this ere 'ospital and lain on that ere trolly; bless me, we all comes ere, we does, and now my boy is out there, and thanks be to God he will be having a good meal at last.'"

### THE RELIEF OF MAFEKING.—AT A FEVER HOSPITAL.

WHETHER cut off from the world or not, no section of people in the country enjoyed the relief of Mafeking more than the inmates of the Grove Hospital, Lower Tooting. It was very shortly after seven o'clock in the morning when the nurses and some of the officials came out on to the large flat roof of the engine-room, and danced and waved flags and sang "God Save the Queen," till they set fire to the patriotism, not only of the neighbourhood, but of the little children inside the hospital. It sounded very pretty to hear the tiny treble voices rising high in the songs and the cheering. Later on in the day a host of flags and a large portrait of Baden-Powell, surrounded with rows of electric light, gave quite a festive appearance to the substantial looking building which offers shelter in time of need to so many thousands.

### THE RELIEF OF MAFEKING.—AT A LUNATIC ASYLUM.

"AN Asylum Nurse," who thinks that some of our readers may like to hear how the "relief of gallant little Mafeking" was celebrated by patients and nurses in one of the small Scottish asylums, writes: "Our people have taken a great interest in the news ever since the war was begun, and when this last success was announced our enthusiasm knew no bounds. We did not boast a British flag, but a messenger was quickly despatched to the nearest village for the necessary materials, and meanwhile an 'impromptu' flag was manufactured from scraps found in the 'fancy dress' cupboard. This was carried to the top of the house, and amidst loud cheering and much scrambling, was finally hoisted in a false chimney pot. 'Tell it not in Gath,' or, rather, let it not come to the ears of the Board of Lunacy, but there were others, besides members of the staff, in that elevated position. Moreover, some windows, I fear, were 'unchecked.' But it is not every day we have a 'relief of Mafeking.' Several people expected that we should have a dance to celebrate the event, but matron thought that would not be 'general' enough. Eventually we decided, as the weather was so fine, to have an 'al fresco' tea party, and invite the gentlemen to tea with us. Accordingly, as soon as lunch was concluded, matron and some of the lady patients, robed in white aprons, set to work and made sandwiches, cut cake, &c., for between 70 and 80 people, nor did we forget a plateful of goodies for each sick-room (male and female). Everyone was anxious to help, even the melancholics seemed to throw aside for a moment or two the terrible cloud of sadness and depression which swamps their poor brains, and to enter into the prevailing joy. The table was spread on the lawn in front of the house, and prettily decorated with red, white, and blue flowers. A *Graphic* picture of 'B.-P.,' wreathed in laurel leaves and festooned with ribbons, was given a prominent place. Small flags were everywhere, and many wore the little medallion portraits and the national colours."

### THE PROGRESS OF THE COLONIAL NURSING ASSOCIATION.

MRS. FRANCIS PIGGOTT, the founder of the Colonial Nursing Association, must be congratulated both upon the success of the fourth annual meeting, and also upon the nature of the fourth annual report. Earl Grey presided, and of the speakers at Stafford House on Wednesday afternoon the Bishop of Ripon represented the Church, General Sir George White the Army, and Sir Squire Bancroft the Drama. Royalty, in the person of the patroness, the Princess Henry of Battenberg, was to have given further *éclat* to the proceedings, but Her Royal Highness accompanied the Queen to Scotland on Tuesday. As to the work of last year the most important items are that the association has broken fresh ground in East Griqualand, Dominica, Tokio, and Singapore; that three nurses have been supplied to a new nursing home at Sierra Leone; and that the number of candidates selected for Government employment has increased considerably. Cape Coast Castle, Old Calabar, Northern Nigeria, Southern Nigeria, and Singapore have all been supplied with nursing staffs by the association. We hope that many who read the account of the meeting, and learn that there are still a number of places abroad which are quite unable to provide nursing for their sick and suffering without help from the Mother Country, and yet badly need it, will become subscribers to this most admirable organisation.

### NURSING AT PEKING.

Last week we referred to the difficulty experienced by the authorities of the Jubilee Hospital at Peking in respect to nursing. It should be added that Nurse Bourgignon, on whom the whole burden of the nursing was thrown for a time, proved herself extremely efficient and assiduous under very trying circumstances. Miss Bourgignon was trained at Charing Cross Hospital, and was formerly Lady Rothschild's nurse at Tring. If a colleague of equal ability can be secured by the Peking Hospital, the committee will have every cause for congratulation.

### THE NURSE DIFFICULTY AT PLYMOUTH.

The difficulty of obtaining nurses is felt, among other places, at Plymouth. At a meeting of the Court of Guardians last week, Mr. T. C. Holland said that two nurses were now leaving, which made five in less than five months, and he naturally assumed that there must be some reason for this constant departure. He complained that nurses were appointed without seeing them, a remark which elicited from Mr. Murray the rejoinder that Mr. Holland imagined that nurses "were sold to the Guardians, and bound hand and foot." This comment appears to have been quite uncalled-for, but there is force in the explanation given by Mrs. Simpson of the state of affairs described. She thinks that the dissatisfaction of the nurses is due to the fact that they have to lodge outside the infirmary, which they consider adds materially to their day's work, and she believes that if good accommodation could be provided inside the "house" there would not be such frequent changes. One thing at least is certain; the clerk to the Court of Guardians ought to be permitted to give the applicants for appointments full information respecting the conditions. This, he states, he has not hitherto been allowed to do, and the nurses engaged have hitherto come to Plymouth without the knowledge that rooms were not provided for them on the premises.

### THE NORTH LONDON NURSING ASSOCIATION.

The pretty nurses' home of the North London Nursing Association, Holloway Road, was a centre of attraction on Wednesday and Thursday last week. The occasion was a sale of work in aid of the funds of the society. The president of the ladies' auxiliary, Mrs. Cohen, received Mr. George Bartley, M.P., who performed the opening ceremony. After Mrs. Powel, secretary of the Executive Committee, had said a few words of welcome and thanks, a tiny boy patient presented her with a bouquet on behalf of the lady stallholders. The nurses themselves found time in the midst of their duties to lend a helping hand in organising the sale, the financial results of which were very satisfactory. On the first day the proceeds amounted to £100. The Association, thanks to Mr. Cohen, M.P., also received handsome cheques from two of the City companies. It is three years since a similar effort was made in its behalf though it is often pressed for money in the pursuance of its admirable work among the poor of North London.

### THE HOXTON SETTLEMENT.

On Friday afternoon, June 22nd, there will be an interesting gathering, under the auspices of the Federated Women's Settlements, at the Essex (Lower) Hall, Essex Street, Strand. The members and associates of the Hoxton Settlement and their friends will have the pleasure of hearing Miss Wald—who will be accompanied by Miss McDowell and Miss Waters—describe the fourth year's training in New York, part of which is now taken in the Nurses' Settlements, New York, to which these ladies belong. Another very interesting item is that the North Hackney High School girls raised £70 for the Hoxton School nurse by an entertainment and sale of work. Miss Honnor Morten and her colleagues deserve the support of the philanthropic public.

### TIRED NURSES AT CHURCH.

The people of Newcastle-on-Tyne do not appear to show the same consideration to nurses at church which is generally manifested. Last week a correspondent mentioned how Canon Gore had enabled a couple of nurses to secure good seats in Westminster Abbey, and it is well known that at a Glasgow church, which is always crowded, nurses in uniform are provided with places whether they are early or late. At Newcastle, however, on a recent occasion when Dr. Watson was preaching at Jesmond, one nurse in uniform stood throughout the whole of the service, and two others left the church because they were too tired to continue standing. This is an incident which reflects discredit upon men who remain seated without giving a thought to tired women who never obtain a full "day of rest" except when they are away on their short summer holiday. But we think that this must be an isolated instance of inattention, for, as a rule, men are very ready to observe courtesy to ladies who are obviously members of the nursing profession.

### SHORT ITEMS.

Visitors to the Woman's Exhibition at Earl's Court who are interested in the North-Eastern Hospital for Children should inspect "Picturesque England," where a representation of the cot now being founded in perpetuity by the Children's Salon may be seen.—At the annual meeting of the Sutton-in-Ashfield Nursing Association it was stated that there are now over 1,300 subscribers, as compared with 207 during the first year's work of the organisation.—Miss Ethel Andrew, whose appointment to Bolton Infirmary was recently announced in our columns, has had to relinquish the post in consequence of being called up by the Army Medical Department to Netley Hospital.

### Lectures on Nursing for Probationers.

By E. MacDowel Cosgrave, M.D., &c., Lecturer to the Dublin Metropolitan Technical School for Nurses.

### VI.—THE BRAIN AND NERVES.

The greater part of the skull is filled by the brain, a large organ weighing over three pounds, and composed of nerve cells and fibres. The brain is divided into two parts—the cerebrum or greater brain, and the cerebellum or lesser brain; both parts are nearly divided by deep fissures into a right and left half, so the terms right and left brain are sometimes

Fig. 12.—The Human Brain.
*a*, cerebrum; *b*, cerebellum; *c*, medulla oblongata; *d*, spinal cord.

used. The brain is covered by three membranes: outside is the dura mater, a strong fibrous layer that lines the inside of the skull; inside is the pia mater, a fine membrane richly supplied with blood-vessels; it is closely connected with the surface of the brain, and conveys much of its blood supply. Between these two membranes is the arachnoid, a double membrane, like that lining a joint; one layer lines the inner surface of the dura mater, the other covers the outside of the pia mater; there is a little fluid between the two layers.

The surface of the cerebrum is divided into a number of folds or convolutions by deep depressions into which the pia mater, with its blood-vessels, dips. The outer side of the convolutions, as well as the walls of the depressions, are covered by a layer of grey matter; the interior of the convolutions and the central mass of the brain are composed of white matter. By this arrangement there is a large surface of grey matter, and it allows of a large number of white fibres ending in the grey matter.

The grey matter is composed chiefly of nerve cells—the highest kind of nerve material. It is the seat of the intellect, emotions, and will, and in it all thoughts, wishes, and impulses arise. The white matter is composed chiefly of nerve fibres, which conduct rather than originate; these keep the different parts of the brain in touch with one another and with the rest of the body.

The cerebellum is situated in the lower part of the skull, under the hinder part of the cerebrum; it only weighs about five ounces. The surface is marked by numerous furrows, which divide it into ten or twelve plates.

The cerebellum seems to have a regulating function, enabling the two halves of the brain to work in concert, and making the different muscles work so as to produce harmonious movements. The fibres from the different parts of the brain run together and pass on to form the medulla oblongata, which is the upper enlarged part of the spinal cord, and lies within the skull. The medulla is the best protected part of the nervous system, lying as it does on the floor of the skull over the dorsal processes of the vertebræ. The medulla is the most vital part of the nervous system, regulating, as it does, such important processes as respiration, circulation, and swallowing.

The spinal cord passes out through the large opening in the occipital bone, and runs down the spine, passing through the rings behind the bodies of the vertebræ. It is covered by dura mater, continuous with that which covers the brain, but the cord does not nearly fill the bony canal, and the membrane is separated from the bones by loose connective tissue, which preserves the cord from injury during bending of the spine.

The cord, like the brain, has grey and white matter, but the grey matter is inside, and the white outside. In a cross section the grey matter is seen to be arranged in shape somewhat like the letter H; two narrow processes of grey matter going to the surface at the front, two shorter and thicker processes being at the back.

Spinal nerves are given off from the spinal cord, passing out to right and left between the vertebræ. Each has two roots, which are connected with the processes of grey matter; the anterior roots contain motor fibres, the posterior roots sensory fibres. If, for example, a hot object be picked up, the sensory fibres convey a message of suffering to the brain, which then influences the motor nerves; the muscles are relaxed and the hot object dropped. The nerves from the spinal cord go to supply the body and limbs. Some of the nerve fibres in the skin end in little oval bodies, which are the organs of touch. The other special senses—sight, hearing, smell, and taste—depend upon nerves which start from the brain. Other nerves from the brain supply the muscles of the face, &c., and some go down to the organs of circulation, respiration, and digestion, and play an important part in the regulation of these functions.

Fig. 13.—Section of Spinal Cord, with Roots of Spinal Nerves—Front View.
1, 1, sensory root; 2, 2, combined nerve trunk; 3, 3, motor root.
(N.B.—No enlargement or ganglion should appear on the anterior root, only on the posterior.)

In addition to the nerves given off by the brain and spinal cord, the body contains Sympathetic Nerves, so called because they were thought to produce a sympathy between the affections of distant organs. This system of nerves consists of a series of small masses of nerve matter, called ganglia, connected by cords of nerve fibres. These ganglia lie on each side of the vertebral column from the skull to the coccyx.

The sympathetic nerves regulate the size of the blood vessels, allowing them to dilate when more blood is wanted by a part; they also regulate the action of the heart and the movements of the walls of the digestive canal.

The sympathetic ganglia are connected with the spinal nerves, and it is through this connection that the sympathetic nerves receive impulses from the brain and spinal cord; for the sympathetic system cannot of itself originate anything.

### Across the Seas.

### NURSING IN CYPRUS.—II.

By a Correspondent.

The nursing staff on my arrival in the colony consisted of two Greek girls and one old man. To these were added two years later, in consequence of the increased number of patients, another Greek girl and two men. The girls wore blue serge dresses (which I at once changed to washing cottons), mob caps, and bib aprons; the men wore white drill uniforms. In addition we had an excellent cook, a laundress, and an odd man, who acted as porter, errand boy, &c.

A gang of six convicts, guarded by a zaptieh, came every morning from six to ten to scrub floors and keep the grounds in order. They also had to pump up sufficient water for the day's use, as we had no pipes laid on. They did not get through much work, as if one of them happened to forget a brush or broom the whole gang had to leave what they were doing and go and fetch it together; consequently, most of their time was spent in parading up and down.

### Our Correspondent's Quarters.

My quarters were in the compound, but a good distance back from the hospital and the kitchen. It was a funny little house, looking like a match-box stood on end. It contained four tiny rooms; one of them on the ground floor was furnished with a divan, table, and cupboard. The cupboard contained a few plates and 24 wine-glasses; not a sign of a teapot or cups and saucers, and I could not procure them in Nicosia. So for many weeks my tea was made and drunk out of a basin. The room above was my bedroom. These were quite sufficient for my wants, and I soon made them quite cosy and homelike, in spite of the missing tea equipage; but I did not like sleeping so far from the wards, as we often had very serious cases and no regular night nurse. I therefore asked and obtained permission from the chief medical officer to utilise an empty room in the central block as a bedroom. My food was cooked in the hospital kitchen and sent across to my quarters, getting very cold in the transit and occasionally shipping rain-water as an addition to the gravy; but I could hardly complain, as the poor patients suffered in the same way. I tried hard to get a covered way built from the wards to the kitchen, but though the Government recognised the need of one they were unable, through lack of funds, to comply with my request.

### The Patients.

My patients were chiefly Greeks and Turks of the humbler class. Of the two I infinitely preferred the Turks; they were cleaner, quieter, more truthful, and in every respect better behaved, and braver too. A Greek as often as not would cry like a baby over the merest scratch, while a Turk would bear a frightful amount of pain without flinching. They all objected to being chloroformed, so except in very serious cases, all operations were done without it.

The zaptiehs (police force) formed a separate class of patients by themselves. They were composed of both nationalities, and I had a great deal of trouble with them at first. They were terrible malingerers, especially the recruits, so the Government ordered that they should pay three and in some cases four and a-half piastres (nine go to a shilling) a day while in hospital, and as they were continually quarrelling with the other patients and escaping from hospital in the night, the female ward was given up to them, and a day and night nurse were appointed from the force to look after them. They were a fine-looking set of men, and most obedient to the doctor's orders, but I could not for a long time get them to obey me. "What! obey a woman, who had no soul, and was no better than a dog? Not they." By patience and perseverance, however, I eventually managed to subdue them; in fact, they became my best patients, and often used to come and see their yatrina (lady doctor) long after they left hospital, bringing me presents of flowers and fruit.

### Difficulties in Nursing.

Another difficulty I had to encounter with the Turkish patients was their aversion to stimulants. The Koran forbids it, so, unless it was disguised with drugs or in an egg-flip, they would not touch it. A jacket poultice was ordered for an old zaptieh one day, and I had to peel off ten vests, some of them quilted, before I could get at him. He was awfully angry with me, and not all the blankets and hot-water bottles I showered on him could convince him that it was not my intention to freeze him to death. Most of our cases were surgical, and they usually did wonderfully well. Patients used to come from all parts of the island to be operated on for stone. I had quite a big collection of calculi; as many as seven, each about the size of a small marble, being removed from one man.

Every Saint's day (and the orthodox church has about one every week, if not more) was a public holiday. All shops were closed, and everyone set to work to enjoy themselves. These festivities generally ended in being turned out of bed in the middle of the night to attend to knife wounds. In spite of the law, which forbids it, every man and boy in the island carries a knife, which is drawn on the slightest provocation.

### No Resident Surgeons.

We had no resident surgeon, so I attended these cases myself, with the help of the dispenser. If I thought there was anything serious I sent a line to the doctor who lived in the town asking him to come over. They were seldom beyond my skill, but once I had a really bad case and could not get a doctor anywhere. One had been sent into the district to investigate a murder, one was on leave, and the third was attending a bad case in town, so I had to set to work and do the best I could.

### A Camel Bite.

It was a bad camel bite. The camel had seized a man by the arm and walked him round the village. It had to be stunned before it would let go. The state of the arm can therefore be imagined. The bones were broken in several places, and it was bleeding profusely. The patient was in a state of collapse, and there was not much time to lose, so I cleaned the wounds thoroughly, picked up a few arteries, and put the arm up on a Stromeyer's cushion. The patient's temperature next day was over 105, and he had every symptom of blood-poisoning; the arm got worse, gangrene set in, and the doctor wished to take it off, but being a Turk the patient would not allow it. The Turk believes that no mutilated person is permitted within the gates of paradise, so he prefers death to mutilation. We dressed the arm twice a day for weeks, and removed bits of dead bone. It got a little better, but I did not think it would be of much use to him. However, one fine day Mr. Turk got tired of hospital, and walked himself and his arm off. The next time I saw him he was perched on the top of a camel, and waved his arm vigorously at me. It was to all intents and purposes as good as the other.

### Climatic Conditions.

The climate of Cyprus is not supposed to be healthy, but it suits some people. The summer months are very hot and dry. All doors and windows are kept closed during the day, and only opened at sunset. In this manner the rooms remain comparatively cool. A certain amount of fever prevails in the plains, and I came in for a good share of it, but if you can manage to get to the hills in the hot season you run less risk of fever. The winter is not cold enough for snow or ice, but fires all day are necessary.

February, March, and April are delightful months. Everything is fresh and green after the rains, whole fields are covered with narcissi, jonquils, and anemones, and the air is perfumed with orange blossom, violets, and jessamine; the violets particularly are magnificent—they are the Neapolitan variety, and are as big nearly as a two shilling piece. I wanted some for a wreath one day, and for eighteen pence got a huge basketful. The Greeks make jam of them, and very nasty sickly stuff it is too. Fruit there is in abundance, and of the very best. Grapes—black and white—apricots, figs, melons, loquats, plums, cherries, oranges, and lemons. Adjoining the hospital grounds there is an immense garden belonging to an old Turk. I used often to wander in, eat as much fruit as I wanted, and carry home a quantity in my apron. The old man was quite satisfied if I gave him a piastre for the lot.

WINE CHEAPER THAN WATER.

The lower classes live chiefly on black bread and salted olives; sometimes a salad is added. Tomatoes, cucumbers, and lettuce are both plentiful and cheap. Olive oil is one of the chief productions of the country, and vinegar is manufactured, too. A bottle of the rough native wine costs something less than half a piastre, so on the whole the poor do not fare badly. One very good grape year, I was told that wine was so cheap that villagers were using it for mixing mortar; it cost less than water. The drought is terrible in the summer. The biggest river in the island flows just behind the hospital. There is not much "flow" about it for several months, only a few shallow pools here and there. When the rains begin it comes down from the mountains with a rush; what has been for several months a dry watercourse is transformed in a couple of hours into a seething torrent.

### The War in South Africa.

### A SCORE OF NURSES TO LEAVE AT ONCE.

THE Secretary of State for War informs us that twenty more members of the Army Nursing Service Reserve are about to leave for South Africa. Of these, five—Misses A. A. Bousfield, M. Dempster, R. Moody, M. A. Rickards, and L. Schroder—will embark at Southampton in the mail steamer on Saturday; and the remaining 15—Misses F. Bishop, A. Cameron, A. F. Clarke, G. Chinnery, J. E. Dods, M. M. Horder, E. M. King, K. E. King, M. R. M. McDowell, N. E. Newton, E. C. R. Philp, W. M. Pooler, H. Swain, A. S. Wyatt, and P. Young—about the 28th, in the "Lismore Castle." Miss Agnes Amelia Bousfield was trained at the General Infirmary, Leicester, and at St. Bartholomew's Hospital. She has since been staff nurse at the East Suffolk Hospital, Ipswich, and the General Hospital, Birmingham, and sister at the Kidderminster Infirmary. Miss Annie Cameron was trained at the Western Infirmary, Glasgow, where she was subsequently staff nurse. She has since been attached to the Nurses' Institute at Edinburgh, and has been district nurse at Carracham. Miss Mary R. Magill M'Dowell was trained at the General Infirmary, Sunderland, and afterwards served as staff nurse. She was then attached to the Hanover Institute, subsequently becoming sister at the Orthopædic Hospital, Dublin. Miss E. C. Russell Philp was trained at the Royal Infirmary, Edinburgh. She has since been successively night nurse, assistant nurse, assistant night superintendent, assistant staff nurse, and head nurse in the same institution. Miss Winifred Mary Pooler was trained at Grimsby and District Hospital. She has since been staff nurse at the Jaffray Hospital, Birmingham; the Royal Infirmary, Bristol; private nurse at the Nursing Institute, Torquay; staff nurse at Cheltenham Fever Hospital; and Queen's District Nurse, Rawtenstall. Miss Helen Swain was trained at Berks Hospital, Reading, and has since been first sister and then night superintendent at the General Infirmary, Worcester.

### Appointments.

BIRMINGHAM INFIRMARY.—Miss E. A. Gittins has been appointed Assistant Matron. She was trained at the infirmary, and holds a first-class certificate and the diploma of the London Obstetrical Society. After her three years' training she was appointed sister of a ward; later, she became sister in charge of the maternity wards, and a year ago she was selected to fill the responsible position of home sister. Miss Gittins has been for seven years a member of the nursing staff, and her appointment as assistant matron has given great satisfaction to all the members of it.

PEMBROKESHIRE INFIRMARY, HAVERFORDWEST.—Miss Ada Britten has been appointed Nurse Matron. She was trained at Hertford General Hospital, and has since been staff nurse for fifteen months at the West London Hospital, lady superintendent for one and a-half years at Andover Cottage Hospital, and matron for five and a-half years at the Southend Victoria Hospital.

KINGSBRIDGE AND DISTRICT COTTAGE HOSPITAL.—Miss Edith Please has been appointed Nurse Matron. She was trained at St. George's Hospital, London, where she has since been charge nurse. Subsequently she was night superintendent at Hampstead Workhouse Infirmary, and Queen's nurse at Neath.

MIDDLESBROUGH WORKHOUSE INFIRMARY.—Miss Rose Wells Pascoe has been appointed Superintendent Nurse. She was trained at King's College Hospital and Queen Charlotte's Hospital, London, and has since been night superintendent of nurses at Fulham Infirmary.

WHITWORTH HOSPITAL, DARLEY DALE.—Miss Sarah Davidson has been appointed Nurse Matron. She was trained at the North Staffordshire Infirmary Union Workhouse, Newcastle-under-Lyne, and has since been nurse at the Royal Infirmary, Darley.

### Minor Appointments.

ST. GEORGE'S INFIRMARY, FULHAM ROAD.—The Misses Emma Nesling Brown, Kate Mary Carter, Emily Malins, Ada Parks, Minnie Penn, Ida Mary Rust, and Adelina Agnes Trotman have been appointed Charge Nurses. Miss Brown was trained at St. George's Infirmary. Miss Carter was trained at the Mill Road Infirmary, Liverpool, where she was afterwards charge nurse; subsequently she was charge nurse at the Sheffield and Prescot Union Infirmaries, and the City Fever Hospital, Liverpool. Miss Malins was trained at the Leeds Union Infirmary, and was subsequently charge nurse at the City Fever Hospital, Liverpool. Miss Parks was trained at St. George's Infirmary, and was subsequently staff nurse at St. William's Hospital, Rochester. Miss Penn was trained at Bradford Infirmary; she has since been charge nurse at the Weymouth Hospital, and sister at the New Infirmary, Wakefield. Miss Rust was trained at St. George's Infirmary. Miss Trotman also was trained at St. George's Infirmary.

WALSALL WORKHOUSE INFIRMARY.—Miss Annie Easton has been appointed Superintendent Nurse. She was trained at the Manchester Royal Infirmary, and has since been night superintendent of nurses at the Workhouse Infirmary, Hoxton, and head nurse at Greenwich Union Infirmary.

CITY HOSPITAL (SOUTH), LIVERPOOL.—Miss Lilian J. K. Dawson has been appointed Charge Nurse. She was trained at the Meath Hospital and County Dublin Infirmary, and the Cork Street Fever Hospital, Dublin; and has since been sister in the Meath Hospital, Dublin.

PLYMOUTH WORKHOUSE.—Miss Sarah Lewis has been appointed Assistant Nurse. She was trained by the Meath Workhouse Attendants' Association at the Crumpsall Infirmary, Manchester.

MIDDLESBROUGH WORKHOUSE INFIRMARY.—Miss Mary Myers has been appointed Charge Nurse. She was trained at Burnley Union Infirmary.

### The Colonial Nursing Association.

ANNUAL MEETING.

The annual meeting of the Colonial Nursing Association was held, by permission of the Duke and Duchess of Sutherland, at Stafford House on Wednesday afternoon, Earl Grey, in the absence of Lord Loch from indisposition, in the chair. The executive committee presented their fourth annual report, from which we take the following extracts:—

Number of Nurses Employed.

The total number of nurses now at work amounts to 56, of which 34 are employed in Government hospitals and 22 as private nurses. The local committees in Mauritius and Bangkok, where the work of private nurses has proved invaluable, have severally applied for a third in each place, in addition to those already employed. In Perak a second nurse has started private work, making with the hospital nurse a total of three European nurses in that place, and a lady superintendent has been sent to the Hatton Nursing Home, Ceylon. The committee feel that the increase in the staff where the experiment has been successfully tried is a highly satisfactory feature of the report.

Breaking New Ground.

Since the last report the following places have been supplied for the first time with private nurses, the association assisting financially where local funds were insufficient: East Griqualand, Dominica, Tokio, and Singapore. At Sierra Leone, Dr. Prout in July last opened a nursing home with the assistance of the Imperial Government, aided by a guarantee from this association of £100 per annum for two years. Three nurses have been supplied to this home, and the reports received state that good work has been done and many lives saved. It is encouraging to note that nine nurses have thus been added to the staff of the association. The number of candidates selected for Government employment has also increased considerably. Cape Coast Castle, Old Calabar, Northern Nigeria, Southern Nigeria, and Singapore have all been supplied with nursing staffs. The Government in each case bears the entire cost of these nurses, who are solely for hospital service.

Tropical Training.

An interesting development of the work of the past year has been the inauguration of a course of supplementary training in the schools for tropical diseases at Greenwich and Liverpool. Six nurses have been through the course, and have all obtained appointments, three on the West Coast of Africa, two at Singapore, and one in Dominica.

The Scottish Branch.

A branch of the Colonial Nursing Association has been started in Scotland to work under the Parent Association, with Lady Balfour of Burleigh as president and Mr. A. A. Gordon as hon. secretary. In addition to enlisting support and collecting funds for the work, it has been arranged that the Scottish branch shall form a nursing sub-committee to interview nurses in Edinburgh, and thus save unsuitable candidates the journey to London. This applies to the preliminary interview only, as the final appointment of nurses rests, as heretofore, in the hands of the London committee.

Raising of Permanent Fund.

Mrs. Chamberlain, in pursuance of the purpose announced in the last report of the committee, made, in May last, a public appeal for funds to raise a substantial sum for investment, and mainly through her personal exertions, which this committee cannot pass unnoticed, the sum of £2,400 has already been collected and invested. It is hoped that this sum may this year be further augmented, £5,000 being the amount to which it is desired to raise the invested capital of the association. With reference to the investments which have already been made, the Executive Committee wish to call attention to the obligation of the association to the Duke of Bedford and the Earl of Selborne, who have been good enough to consent to act as trustees of the association, together with the president and the hon. solicitor.

The adoption of the report was moved from the Chair and seconded by Sir Squire Bancroft, who has done so much for philanthropic undertakings in giving recitals in England and the colonies.

The Bishop of Ripon said that young Englishmen going out to the colonies and elsewhere had to face strange vicissitudes of climate and illness, sometimes of illness unknown to Europeans; and it was a necessity for this new condition of things that nurses should be trained to anticipate the symptoms of these strange illnesses. Almost everyone had heard of cases of mistaken diagnoses, owing to the physician not being acquainted with the conditions which led to the illness, and this would be largely counteracted by the presence on the spot of nurses trained in the way he had sketched. It was the duty of those at home to realise that wherever Englishmen go everything should as far as possible be provided to meet these conditions of climate and disease. The word "Colonial" seemed to have been almost blotted out by recent events; the chivalry, devotion, and the heroism of the Colonial troops in the present war seemed to wipe out the distance separating them from the mother country. He appealed to the audience to enable the Colonial Nursing Association to maintain this British principle of self-reliance—this old fashioned spirit, which permeated all parts of the globe where the English tongue was spoken, this gift of Almighty God. Some, the Bishop added, were too ready to play the part of nurse—they were not the nurses for whom this association existed. We did not want ladies who fed enteric fever cases with buns; what we wanted were trained women, the kind of women who should be the living accompaniments of all Imperial movements. They were a living force, the rest were only a name.

Sir George White, who was greeted with prolonged applause, said that he had just come from witnessing the courage and endurance of some of the finest men it had ever been his privilege to know. His experience extended not only to the Colonies, but to that brightest jewel in the Queen-Empress's crown—India. When he went first to India, 25 per cent. of the army were carried off by cholera in a very few days. Now, this heavy loss from the dread disease has almost entirely disappeared. He had only known one instance of such a loss, of late years, and that, in his opinion, had been caused by a want of inquiry at the beginning. Old-fashioned filters were used, and these, with the idea of precaution, were carried by the troops when the camp was moved. A component part of the filter was sand, and this had been taken from the bed of a river largely used by pilgrims who were infected with the disease. Enteric fever was now the scourge of our army in India, and of all diseases this most required the presence of trained nurses. He had just come from Natal, where among his own force were at least 11,000 cases of enteric, besides other fever cases, which had the fever temperature, but of which the doctors could not be certain at an early stage what form the fever would take. General White appealed to the audience to consider what an enormous advantage it would be, in their own case, if, say, a child were ill abroad to know that an institution existed close by to which application might be made for a trained nurse; and he instanced the case of a lady, well-known in London society, who had sustained great loss in this present war, who, in Simla, was unable to obtain a nurse in case of necessity. Nurses were there, but they were attached to institutions, and were not available outside. Sir George White concluded by saying that he could not imagine any better or more deserving form of charity than the Colonial Nursing Association.

Other speakers were Sir Henry Norman, who proposed the re-election of officers; Mr. Dalton, the association's solicitor; and Lord Westmeath, who seconded the vote of thanks to the Duke and Duchess of Sutherland, Lord Grey, and the other speakers.

### The Nurses of the Imperial Yeomanry Hospital.

WRITING from Deelfontein under date of April 19th, a correspondent says: "Since my last letter we have made a good many strides forward towards the entire completion of the hospital. The huts which I described to you then were built by a Cape Town firm, and they are all now finished and occupied, but in addition to these we have in course of erection some English huts, three of which are complete and ready for occupation. These will ultimately be nine in number. They are much larger and wider than the Cape Town huts, and have accommodation for 34 patients each. They look very bright and homelike with their beds covered with scarlet blankets. Down the centre of the wards are long tables on which stand writing materials, doctor's jug and basin, and vases of flowers, exactly like our own English hospital wards at home. One of these huts, *i.e.*, the Sherwood Rangers', is set apart entirely for officers. It is built in three divisions, the middle division being again divided into sisters' room and patients' convalescent room.

### THE ENTERIC CASES.

"We have now such a large percentage of enteric cases sent down to us from Bloemfontein and elsewhere that an entire block of huts has been put apart for their use. All appliances, lavatories, &c., are separated from the rest of the camp, and the drainage has been so arranged that there can be no fear of any contamination to other parts of the camp. We are getting quite used to a whole train-load of patients coming in at a time, and often before we can receive fresh cases we have to send a batch of convalescent men down to Cape Town to make room for the newcomers. The Red Cross train, as you know, perhaps, is fitted up like a ward on wheels, and two sisters, in addition to a medical man, are always in attendance on the patients. At the present time we have all our beds, numbering 500, full; the last large contingent of sick and wounded came from Bloemfontein, some of the men having been wounded at Sannaspoort, near the Waterworks. A number of men belong to the R.H. Artillery and Roberts' Horse. None are very seriously damaged, but most of them agree that they 'have had about enough of it for the present.'

### DESCRIPTION OF A FIGHT.

"A sergeant of the R.H.A. describes very vividly the fight in which he took part at Sannaspoort. He says . . . 'We had to advance for a drift against a house flying a large white flag (the latter signal showed that the inmates were loyal to Britain and unwilling to fight against her). Our envoy had halted this side of the drift, and the U Battery R.H.A. trotted up alongside of the drift and dismounted. My battery halted, and just then a bullet whizzed over my gun, but no one seemed to realise where it came from. We received orders, "Sub-division, left wheel," and we went at a walk in our new direction. We had only proceeded about three hundred yards when we got a volley from our right, which whizzed over our heads like a hailstorm. The order then came, "Sub-division, left wheel, gallop," and away we went like mad up the plain like a flash of wind, and came into action about 1,000 yards from the enemy. We gave them a few shrapnel shells, but it was awful the way our poor gunners kept falling down, shot. The officers also soon became scarce, but we kept up the fire until the officer in charge of my section got hit, and I had to take his place.'

### HIT BY A BOER BULLET.

"'After about a minute's action I felt a bullet hit me in my right knee, and then I in my turn had to fall out. I crawled to a bank and got my wound dressed, and found the bullet had gone right through my left thigh as well. Directly after I was shot the order came for the battery to retire, and the men who were left, helped by officers, men of Roberts' Horse, and Mounted Infantry, saved the other four guns amidst a hail of bullets from the Boers (who had advanced up to 200 yards range) and galloped them away under the very noses of the enemy. I was in a hut when the Boers came and made us all prisoners of war. We felt very miserable and sad, but they left us until next day, when the Lancers came up and the Boers soon cleared off, but at night-time they started shelling the hospital huts, though they never managed to hit any of us. Our guns started firing at the enemy about 1,000 yards away from the hospital, and when our people began to move us in wagons the Boers immediately commenced to shell the wagons. It was awful to hear the wounded men shouting and groaning as the native drivers made their mules trot over the stones; but, thank God, we got clear of the guns, and when we were out of range of the enemy the drivers slackened speed a bit, and were more careful where they were going. As long as I live I shall never forget March 31st, 1900, in the Bloemfontein Waterworks.'

### THE HAVEN OF REST.

"This will give you a very good general idea of what the majority of our men who have been in action have experienced, though very few will tell you of the hardships, in addition, which they have undergone, such as the long, weary marches, the sleepless nights, the awful thirst, when water, except of the very foulest, is unobtainable, and how for ten days some of them have lived on a biscuit a day and nothing more. All this is forgotten, however, when Tommy finds himself in the haven of rest, as he affectionately calls the hospital. I am sure if you were only present at one of our concerts, which are given weekly, and heard the hearty way in which they join in the chorus of the 'Rowdy, Dowdy, Boys' you would think that they had never suffered a single ache or pain. These concerts are greatly enjoyed, not only by the occupants of the convalescent tents, but by the whole staff.

### THE HOURS OF DUTY.

"I do not remember if I told you that the arrangement here for night and day duty is the same as in the London hospitals, a fair proportion of the sisters being on night duty, which I believe is unusual out here. The night sisters seem to think they have the best of it just now, as they escape the blazing heat of the day, though it is bitterly cold, especially in the very early morning before the sun gets up. We have had no great disaster in the way of sandstorms since I wrote last; but this afternoon a tremendous cloud of locusts passed over the camp, which reminded some of their Old Testament history. We have an English Church chaplain attached to the hospital, and every week the Roman Catholics are attended by a chaplain from De Aar, who is serving the military hospital there as well. On Easter Day the tent used as a chapel was prettily decorated with flowers from the veldt, while for the altar white flowers were specially sent up from Cape Town.

### CRICKET MATCHES FOR THE CONVALESCENTS.

"The patients are well supplied with books, and the library is much appreciated by them. In addition to the concerts got up for the entertainment of the convalescent patients, we have cricket matches, a team coming up from Richmond Road (our nearest station) to play against the hospital eleven. You can gather from this that we manage to make time pass pleasantly, even though we are living in the middle of the Karoo Desert."

### Nursing in Tubercular Disease of Joints.

Clinical Lecture delivered to Nurses at the City Orthopædic Hospital, Tuesday, May 15th, 1900, By Noble Smith, F.R.C.S.Ed., Surgeon to the Hospital.

In my last lecture I stated that you ought to look on all cases of tubercular disease of joints as curable. What I mean is that if any patient suffering from such disease is thoroughly well nursed and the diseased part thoroughly fixed, there are so few who will not get well that the nurse should certainly look for a cure as the natural result of treatment.

We can never be absolutely certain that a patient is cured of tubercular disease, but upon the other hand we need never despair, for, under most unpromising conditions, a cure is possible; that is why I wish you to look upon every case as curable. If you start with this view, and do all you possibly can to help the surgeon you will very rarely be disappointed.

The fact that so very large a majority of cases of caries of the spine get absolutely well and remain well, is positive proof that it is not necessary to excise a joint for the sake of preventing the extension of the disease. We cannot excise the vertebræ, and they get well without excision.

Most of the remarks made at the last lecture upon the care necessary in moving a patient with tubercular disease of the spine apply to the same disease affecting other joints. To fix the hip-joint the whole of the spine and the whole of the thigh must be controlled by the splint, and it is better still if the whole of the leg down to the ankle is included. Thomas's splint is a very thorough support, but as a rule it is made too heavy, and therefore is difficult to bend into the desired shape. We use here a somewhat different form of splint, invented by our late colleague, Mr. Chance. It acts on the same principles as Thomas's, and was in use in this hospital long before Thomas invented his. The knee and ankle joints are much more easy to fix than the hip, and it is much easier for the nurse to move a patient without disturbing these diseased joints than when the hip or the spine is affected.

Chronic abscesses are a serious complication of tubercular joint disease, but if treated energetically and carefully attended to by the nurse, the results are very satisfactory. There need be no fear on the part of the surgeon in opening a chronic abscess if that abscess be treated antiseptically afterwards. All kinds of devices have been adopted for excluding air from the wound after operating, but such care is quite secondary to washing out the abscess with antiseptic lotion. For the last twenty years and more I have used a 1 in 40 solution of carbolic acid for this purpose, and only remember two instances in which there were any symptoms of carbolic poisoning, and they were trivial in character. However, the nurse should constantly watch for symptoms of carbolic poisoning. It shows itself by smokiness in the child's water. If you place a few drops of ink in a wineglassful of water it produces just the same smoky appearance as results from the action of carbolic acid on the excretion of urine.

After opening a chronic abscess, the surgeon is guided by the nature of the case as to the immediate treatment. He may find it possible to scrape out the whole contents of the abscess and close the wound at once, but it is rare that this treatment can be carried out. As a rule, and this is especially the case in abscesses connected with the vertebræ, there is actively diseased bone at the base of the abscess, and so long as this condition remains, that is, until the bone has ceased to ulcerate, there will be little use in closing up the outer opening, because a still further collection of matter is likely to take place. The most satisfactory way of dealing with such a case is by using a daily injection of antiseptic fluid. It is a good plan to commence the injection by using warm water alone until the water comes away clear, then about 2 oz. of a 1 in 40 solution of carbolic can be injected (warmed). It is very necessary that the lotion should penetrate to the deepest part of the abscess; this is sometimes difficult. A syringe holding 2 or 3 oz. or more is convenient. Sometimes there is some obstruction to injection, and the fluid runs out of the wound rather than penetrates into its depths. The best plan is to have a small-sized drainage tube, without the drainage holes, long enough to reach to the bottom of the abscess.

In some cases I have passed a tube to the depth of 14 to 16 inches. When the tube will go no further it is better to draw it back a little, so that the fluid may not be impeded. If this injecting is done gently and carefully, the patient rarely suffers any pain. The tube is only to be used at the time of injection and not as a drainage tube.

When the sinus—that is, the narrow canal which leads from the surface wound to the cavity of the abscess—is tortuous, there may be a difficulty in inserting the tube sufficiently far. There are various plans by which this difficulty can be overcome. For instance, when the tube has been inserted as far as it will go without much force, some fluid may be injected, and the sinus opened out by the fluid, when the tube may be pushed a little further, or the tube may be twisted as it is inserted. It is preferable to fill the tube with the lotion before inserting it into the abscess, so that as little air as possible should be conveyed into the cavity. I do not think that the entrance of air in this way would do any harm, because the action of carbolic acid counteracts the development of bacteria, and a frequent daily application of this lotion is sufficient to prevent harm occurring. However, the absence of air allows the fluid to get to the walls of the abscess more thoroughly.

I can speak most confidently of this method of dealing with these abscesses. I have never hesitated to make a free opening into an abscess pointing in any position, and I have never known anything but a good result to follow. The abscesses quickly heal up, often in a few weeks. In a very few instances it does not heal up so soon, but at the present moment I can only remember one case in which such an absence of a perfect result has followed this treatment. In this case there was a great and rapid improvement until nothing but a watery discharge came away, but this persisted for two or three years, although three other abscesses, two lumbar, and one psoas healed up entirely in the ordinary way.

My explanation of this failure to close up entirely is that there is a large piece of necrosed bone at the bottom of the abscess, and that it acts as a foreign body and keeps up the irritation. One word of caution: be careful not to expose any child too much, or for too long a period. Cover up the patient with a blanket while carrying out the injection.

[These lectures, which are given every Tuesday, may be attended by any nurse.]

### Death of a Belgian Nurse at Pretoria.

It is announced that Madame Laridon, a well-known Belgian nurse, died at Pretoria on Friday from "overwork and grief occasioned by the Boer disasters." Madame Laridon, who had been decorated with the Order of Leopold for previous services on the battlefield, went out to the Transvaal with the Belgian ambulance, and devoted herself entirely to the work of ministering to the sufferings of the Boer soldiers.

### Echoes from the Outside World.

### AN OPEN LETTER TO A HOSPITAL NURSE.

What can I say about the relief of Mafeking? That I failed to sleep for joy on Friday night; that I decorated myself and my establishment lavishly on Saturday; that I seized every opportunity which offered for joining in "God save the Queen" till I was hoarse, and then felt after I had done all that I had utterly failed to show anyone how glad I was? But this is no news, for everyone did the same, and I expect many, too, felt throughout the hours when we realised the relief which had come to us, as well as to Mafeking, that laughter and tears were strangely close together. Till Monday afternoon the haunting dread would recur at intervals, "Suppose it is all a hoax of the Boers," and I heard of many folks, including the Governor of one of Her Majesty's prisons, who refused to allow flags to be hoisted; but at last the War Office was able to set our minds at rest, announcing also that brave Baden-Powell was gazetted Major-General, and quiet contentment took the place of uproarious mirth. Ever since the good tidings reached us, as each meal came round, I wondered what Mafeking had to eat. It is only now that we shall know what the sufferings have been. The following extract from a letter written in Mafeking January 22nd is only a foreshadowing of the details likely to be sent to us during the next few weeks. It was written by the mother of four little girls: "When shall we be relieved? Surely England cannot have deserted us. Hettie and Nellie lie in the cemetery, baby lies in her cot a little skeleton, and as I write my tears fall on the pallid face of Winnie. She is dying." And that was written four months ago!

Upon the occasion of my second visit to the Academy I made a point of taking special notice of the work done by women. The days when flowers and fruit were the only "ladylike" subjects for a woman to take up is past, and I was specially struck with 373, by Hannah Myers. It is entitled, "And wondered at the fair face mirror'd there," and shows a girl in a green meadow, with a forest behind, lying, quite nude, prone upon the ground, gazing at the reflection of herself in a half-stagnant pool. To paint such a picture correctly requires a fine grasp of anatomical drawing, and, though I would never care to hang it in my drawing-room, one feels proud that a woman can perform such a task. Two pieces of statuary, one in bronze and one in marble, also impressed me in the same manner. One is "Fisherman Hauling in a Net" (1,968), the other "The Hammer Thrower" (1,986) by Ruby Levick. In both the man is stripped to the waist and is bending down to exert himself, so that the muscles of the back stand boldly forth. It is strange that a girl should have selected so masculine a subject, but the result justifies the choice. By far the most remarkable production by a woman, however, is "Horses Bathing in the Sea" (427). It is a very large canvas, hung upon the line, and is instinct with life. The horses, as they splash and rear and prance, are simply splendid, one, who threatens to become unmanageable, so that the carter in charge of him has hard work to restrain him, being a wonderful piece of painting. Decidedly those who declare that they see in the artist, Lucy Kemp-Welch, a future Rosa Bonheur, are justified in their high opinion, and I should be really glad to hear that she had secured the £1,000 which she is asking. Two portraits by Mrs. Waller (408 and 484), are very creditable performances, and I was considerably impressed with "The Silent Life," by Dora Noyes. It represents a nun clad in white, wearing a large shady hat to protect her from the sun as she goes down the garden path, ablaze on each side with spring flowers. The face is such as you may see beneath a monastic coif any day, contented and restful, but lacking in brightness, the mouth firmly compressed, and slightly austere, with a faint suspicion of moustache upon the upper lip. Amongst the miniature painters the women, of course, stand out prominently. I specially remarked "Frida' (1,481), by Winifred Marshall, a portrait of a little child standing in a field, with blue strings to her big hat hanging loosely down, and a touch of dull red flower at her feet. Another clever miniature was "Mira" (1,498), by Maud Nye, who paints in a very realistic and original manner. She entirely escapes the sameness of style which is generally noticeable in miniature painting, and makes of her subject, no longer a young woman, a pleasing picture, and apparently a speaking likeness.

Nurses are personally interested in the movement for securing cyclists adequate accommodation and facilities in travelling. It is curious that any of the railway companies should need to be spurred into action when it is so obvious that their interests would be served by catering in the best possible manner for an ever-increasing army of customers. Cycling has long since ceased to be either a fad or even a mere recreation; it has become the necessity of the many. But the traffic by rail is yet only in its infancy, and the companies who exert themselves to cultivate it will reap an abundant harvest. Of course, as the President of the Board of Trade hinted the other day, there must be legislation if the convenience of a very important section of the community continues to be ignored; but the managers of the great lines will prove themselves strangely wanting in the foresight they are supposed to possess if they require to have concessions wrung from them by compulsion which should be gladly volunteered.

As yet the weather has been far too changeable for the wise to sport cotton blouses, but when the sun shines persistently you will find that all the newest are made in the front on the cross, frequently with yokes of a contrasting colour, and the effect is very nice, more especially in bright cottons or muslins. The hideous dresses and hats of real khaki colour have had their day, and as ladies have already proved for themselves how unbecoming the hue is to any but the most brilliant complexion, the new tones are much less aggressive, being more of a fawn shade. Fringed scarves are hung on to all parts of "dressy" garments, tied in bows at the back, arranged from the shoulder to hang down in front, or knotted at the side. Festooned around a morning hat so that the fringe dangles over one ear they are, to my mind, both ugly and incongruous, but not so much so as the fashion of twisting a piece of tulle straight round a sailor hat on the top of the ribbon, not even fastened off with a bow, the whole decoration being suggestive of a new use for a worn-out necktie. One of the novelties of the season for those who have many costumes and are likely later in the season to travel is the sunshade with detachable cover. Each sunshade is provided with half a dozen covers in various shades, some useful, some more fanciful. It takes three minutes to make a transformation from, say, a dark red satin, chiffon trimmed, suitable for a dull day, to a dainty summer shade of figured chiné silk, sweetly soft with lace, the very thing for a fête or a garden party. Most of us, however, find one sunshade a season as much as we require. Off duty times do not come often enough, nor are they sufficiently lengthy for many festivities to be possible to nurses, so I expect that the "Araso," as the last new invention is called, will not be much patronised by the nursing profession.

### The Nurses' Bookshelf.

[We invite Correspondence, Criticism, Enquiries, and Notes on Books likely to interest Women and Nurses. Address, Editor, The Hospital (Nurses' Book World), 28 & 29, Southampton Street, Strand, London, W.C.]

Sick Nursing at Home. By L. G. Moberly. (The Scientific Press, Limited, 28 and 29, Southampton Street, Strand, W.C. Pp. 82. Price 1s.)

This unpretentious little book will no doubt be found of very real assistance to those for whom it is written, viz., those who are called upon to undertake nursing in their own homes. There are many minor ailments that an intelligent woman, aided by such a little brochure as this, can readily undertake to the satisfaction of medical man and patient alike. Though there is nothing new in the maxims laid down, yet they do not pall by repetition, for the simple reason that they are so easily overlooked or put on one side as unworthy consideration by reason of their very simplicity. The rule, for instance, to "have all your appliances about you before you do anything for your patient" is an excellent one, but one too often honoured in the breach than the observance. Again, great stress is laid on the importance of little things in performing offices for the sick. The chapters on food and medicine, poultices, and minor ailments all abound with good practical hints and sound common sense. We must confess taking exception to covering up poultices with mackintosh or gutta percha tissue, neither of which are good things to use under such circumstances; a good layer of cotton wadding or flannel is quite sufficient to keep in the heat, and is much better for the patient. It might have been as well to give some limit as to the time a mustard plaster should be applied, as there is a risk of blistering the skin if left on for longer than ten minutes or a quarter of an hour, and this would probably not occur to the amateur nurse. The book is otherwise eminently practical throughout, and is well qualified to serve the purpose for which it was written.

Life and Happiness. Auguste Marrot. (London: Kegan Paul. Paris: Lilian Fishbacher. 8vo., pp. 90. 2s. 6d. net.)

The opening sentence of the author's preface is true enough, "You will find nothing strikingly fresh in these pages." The volume consists of a few chapters on the general hygiene of the young, and the general principles of moral and intellectual training. There is little to quarrel with in any of the advice, but the advice is so general and so sketchy that only intelligent and highly-principled parents could make practical application of it; while there is nothing in the whole book which would be new to intelligent parents. The most commendable parts of the book are those on overeating and on the fostering of intelligent thoughtfulness in children.

Home Nursing of Sick Children. By J. D. E. Mortimer, M.B., F.R.C.S., L.S.A. (London: The Scientific Press. 1899. Price 1s.)

This little book (one of the well-known Burdett Series of shilling handbooks) will be found very useful by young nurses when they find themselves launched into the world and have to undertake the nursing of sick children in their own homes, often without many of those appliances which seem to come so naturally to hand in a well-equipped hospital, and always without that strange hospital influence which, whether it arise from discipline or from imitation, leads children in hospital to do as they are told, and to submit to what is done for them without complaint in a manner quite different from anything that is at all commonly seen in private practice. After an introduction, in which many hints are given in regard to the management of children, and a section describing what should be looked out for by the nurse in her observation of a sick child, we find sections devoted to such topics as feeding, the administration of nutrient enemata, sleep, bathing, massage, &c., and then we come to the various diseases from which children more especially suffer, after which follow operations, various surgical diseases, and lastly several minor matters are dealt with, such as convalescence, travelling, and sea bathing. Take it altogether we think it will be found an acceptable volume to many, especially to those who in the course of their training have not been able to spend much time in a children's ward.

The Art Portfolio. (Messrs. Jobson and Co., 6, Victoria Avenue, E.C.)

Number 10 of this publication contains four interesting Jacobite pictures. The photogravures are excellent reproductions. The first represents Prince Charlie himself. "Raising the Standard, Glenfinnan" is especially fine, whilst all are worthy of separate framing. The publication is a remarkable production for one shilling.

Two little works, each bearing the title of The Nurse's Report Book, have just come under our notice. They both appear to possess equal merits with regard to arrangement, and will largely depend for preference on the individual taste and requirements of the purchaser. The general scope does not admit of much variety from the nature of the subject. Suffice it to say that both possess points of excellence, and they only differ in the arrangement of details. Miss C. M. Lohr is to be congratulated on having reached a third edition, which is proof enough, if any were needed, of the usefulness and popularity of the "Report Book" which bears her name.

### The "Nursing Notes" Exhibit at Earl's Court.

In the Hospital Section at the Earl's Court Exhibition, which is excellently arranged and full of interest, there is in one corner a case which will appeal particularly to nurses. It is an exhibit of "Nursing Appliances designed by Nurses," collected and shown by the editors of *Nursing Notes*. Many ingenious contrivances, the result of practical experience, find a place there. The Q.V.J.I. is represented by the "Operation Basket" and "District Bag" used by the Queen's nurses. Miss Monk, of King's College Hospital, sends a "Test Case" of her own designing. There are several admirably contrived baskets and cases for district nurses to carry on bicycles, and amongst other appliances may be mentioned an excellent "Ward Box," supplied with every cleansing requisite; a "Bed Cradle," for district use; "Bed Crutches," a "Flap Retractor," and "Drainage Tube Forceps," portable sterilizers, a glass tube for keeping catheter, nurses' wallets, &c. Nurses should make a point of examining this interesting little show for themselves.

### To Nurses.

We invite contributions from any of our readers, and shall be glad to pay for "Notes on News from the Nursing World," or for articles describing nursing experiences, or dealing with any nursing question from an original point of view. The minimum payment for contributions is 5s., but we welcome interesting contributions of a column, or a page, in length. It may be added that notices of entertainments, presentations, and deaths are not paid for, but, of course, we are always glad to receive them. All rejected manuscripts are returned in due course, and all payments for manuscripts used are made as early as possible at the beginning of each quarter.

### Everybody's Opinion.

**[Correspondence on all subjects is invited, but we cannot in any way be responsible for the opinions expressed by our correspondents. No communication can be entertained if the name and address of the correspondent is not given, as a guarantee of good faith but not necessarily for publication, or unless one side of the paper only is written on.]**

### GREENWICH INFIRMARY.

"A WEARY CHARGE NURSE" writes: Having seen a letter from "A Hard-worked Probationer," I should like to say I think she has made a mistake, as the off-time is a church pass every Sunday, two hours one week, and from two till ten p.m. the next. She also forgot to mention the pass we have every evening from a quarter to eight till ten p.m.

### SHOREDITCH INFIRMARY.

"C. R." writes: Two trained nurses having had a short experience in Shoreditch Infirmary complain that the work is very unreasonable, and that a single nurse on night duty has as many as seventy patients to attend to. The Nurses' Home leaves nothing to be desired.

### THE PORTSMOUTH ROYAL HOSPITAL.

The MATRON writes: The following statement appeared in the Notes and Queries column of this week's issue of the "Nursing Mirror": "Of the three hospitals, the Royal South Hants and Southampton Hospital, the General Infirmary, Salisbury, and the Royal Portsmouth Hospital, the first-named only offers three years' training." I beg to say that the training in this hospital has for some years been for three years, no certificate being given for less than that period except in the cases of a few probationers whom we train for two years for a private nursing institution.

### LADY DISPENSERS.

MISS EMILY L. B. FORSTER writes: In your "Notes and Queries" you state that the Apothecaries' Hall "Assistants' Certificate" will not qualify for a post as dispenser, only as assistant dispenser. I am in charge of the Ladies' Department at Westminster College of Pharmacy, and as nearly all our passed students hold appointments as dispenser (in charge of the dispensary) at hospitals, provident dispensaries, and Poor Law, and in every case are the only dispenser attached to the institution, I thought I would draw your attention to it. The diploma is called the Assistants' Certificate, as the dispenser may only dispense a doctor's prescription, and may not sell medicine. We have an advertisement in your paper very often. Of course, we do not publish the appointments our students get, but I could give you the names of many at public institutions.

### THE AGE LIMIT.

"SISTER ELLEN" writes: I find great pleasure in reading your paper, in all its varied news, lectures, letters, &c., and heartily desire to thank Miss Mary Gardner for her defence of nurses over 35 years of age, which especially rouses my sympathy and gratitude. Being myself much past the age mentioned, I feel extremely fortunate in having obtained lately (with little trouble) an appointment as nurse-matron, where, in addition to cottage hospital work, with its numerous duties, I also have district, dispensing, and out-patients to attend, and although a very busy life, I am happy and comfortable, and meet with the greatest kindness all round. As "Pavo" remarks, "A nurse at 35 and over has her judgment, common-sense, and self-reliance matured, and by her wide experience is more comfort and help to patients and friends" than the young ones who are often selected in these days.

### THE USE OF THE OBSTETRIC BINDER.

"M. L." writes: I think your correspondent "Monthly Nurse" somewhat vague in her ideas respecting the obstetrical binder, and also on the cases she refers to. It would be interesting to know something of the history of the labour in each case and the after-treatment independently of the binder, its use being to stimulate uterine contraction and to afford support and comfort to the back, the sacro-iliac sutures of which have undergone severe strain during the passage of the child. In the majority of cases the uterus sinks down behind the pubes in eight or ten days, and is then little, if at all influenced by it. So much in its favour. On the other hand, in many cases I have been obliged to loosen the binder considerably in order to relieve, in some cases cramp, and in others that terrible aching of the legs, that I am fast coming to the conclusion that Dame Nature is all sufficient; of far greater importance to complete involution in my mind is the nursing of the baby. Since 1884 nearly my whole time has been spent in maternity work; in no one case has the doctor mentioned the binder after the first week, neither have I had a case of secondary hæmorrhage.

"L. O. S." writes: No fixed rule can be applied to binders. To begin with, it is best to choose a good hospital for training. There you will get the best obstetrician and best training, and be taught everything worth knowing in the way of nursing. Then, see that you carry out your own hospital rules in your after-work, either district or private. Do not be trying every little thing you hear of. Remember that when you have a doctor in attendance you are there to carry out his orders; but if he sees you know your work he will not give you many. Some doctors like binders, others do not. The patient herself nearly always does; if properly put on they say it is a great comfort. Secondary post-partum is not likely to come on at the end of the second week just because the binder had been discontinued. I hope "Monthly Nurse" will not think I am unkind, but she has either been badly trained or has not carried out her training. I think she requires to know more about post-partum and secondary post-partum. As to the use of the obstetric binder, I like my patients to have one for six or seven days, but I never force them one way or another. If the doctor says, "I do not wish a binder," well, I do not put one on, and there is sometimes a little tact required with the patient; but get to know what his wishes are and do it, he will always tell you his reason, and he is the responsible person. If you are qualified to attend cases (normal) without a doctor you are safe in using a binder; it exerts a gentle and moderate pressure on the uterus and abdomen.

### DIET AND ITS EFFECT ON LABOUR.

"A. R. H.," midwife Q.C.H. and L.O.S., writes: Some time ago a book came under my notice, parts of which were intensely interesting to me, dealing as they did with the unreasonableness of women of the higher grades of society suffering so much more than do their poorer sisters during labour. This book was written by an American lady doctor, and, given anybody who thoroughly understands the subject, and can sift the parts that appear overdrawn, the substance should be an incalculable benefit to womenkind at large, and should be generally made known by nurses who are interested in such cases. The prevailing idea of the book is that women should give up eating meat entirely, and during their pregnancy should live on fish, eggs, and any amount of fruit and vegetables. They should take as much fresh air as possible—deep breathing being very beneficial—and walk as long as such exercise can be borne twice daily. I have had many opportunities of drawing conclusions myself, both in hospital and private nursing, and I think I may say, with hardly a single exception, I have seen that those of my patients who, from inclination alone, lived on fruit and vegetables almost entirely, had abnormally short and little-painful labours. They also got about to the day of the baby's birth with energy and briskness. Those, on the contrary, who lived largely on meat and rich foods, suffered proportionately greatly, the parts being very rigid. In women of maturer age, where rigidity would have naturally been expected, I have found hot sitz-baths of immense benefit, and the easiest labours following them. Now, I do not profess to know anything personally of those nomadic tribes who appear to get babies painlessly by the roadside and continue their journey within an hour or two of the labour, but I must say that in hospital and on district in London the poor women seem to get much quicker and easier labours than do women more blessed with the good things of every-day life, and their diet surely consists hardly, if at all, of meat. It would be interesting to know whether any more of your readers have drawn the same conclusions as myself, and I trust that you may find room in your invaluable paper to insert this.

### For Reading to the Sick.

### ASCENSIONTIDE.

"This same Jesus which is taken up from you into Heaven shall so come in like manner as ye have seen Him go into Heaven."—*Acts* i. 2.

Soft cloud, that while the breeze of May
Chants her glad matins in the leafy arch,
Draw'st thy bright veil across the heavenly way,
Meet pavement for an Angel's glorious march:

My soul is envious of mine eye,
That it should soar and glide with Thee so fast,
The while my grovelling thoughts half buried lie,
Or lawless roam around this earthly waste.

Chains of my heart, avaunt I say—
I will arise, and in the strength of love
Pursue the bright track ere it fade away,
My Saviour's pathway to His home above.

Till resting by the incarnate Lord,
Once bleeding, now triumphant for my sake,
I mark Him, how by seraph hosts adored,
He to earth's lowest cares is still awake.

He listens to the silent tear
For all the anthems of the boundless sky—
And shall our dreams of music bar our ear
To His soul-piercing voice for ever nigh?

Nay, gracious Saviour—but as now
Our thoughts have traced Thee to thy glory throne,
So help us evermore with Thee to bow
Where human sorrow breathes her lowly moan.

—*Keble.*

**Reading.**

The Ascension of our Lord Jesus Christ is a truth upon which it is impossible for a devout Christian to dwell without glowing exultation. It is the sure witness to him of the transitory character of life's ills and disappointments, and the assurance of the ultimate reward of a faithful endurance of those ills. The Ascension of our Lord was His entrance upon His reward after a life of suffering and apparent failure. "Behoved it not the Christ to suffer these things, and to enter into His glory?" To believers in the Ascension the encouragement is given, "Let us run with patience the race that is set before us, looking unto Jesus, the author and perfector of our faith, who for the joy that was set before Him endured the cross, despising shame, and hath sat down at the right hand of the throne of God. For consider Him that hath endured such gainsaying of sinners against themselves, that ye wax not weary, fainting in your souls." Belief in the Ascension, and all that is involved in that mystery, aids us to estimate aright all forms of earthly suffering, disappointment, and loss. It enables the righteous man, face to face with trials of every description, to say, "What does it matter, it will not last, there is peace and glory beyond." "For I reckon that the sufferings of this present time are not worthy to be compared with the glory that shall be revealed to usward."—*From "The Practical Religion," V. Staley.*

When Christ went up to Heaven the apostles stayed,
Gazing at Heaven, but their wills on fire,
Their hearts on flight along the track He made,
Winged by desire.

Their silence spoke; Lord, why not follow Thee?
Home is not home without Thy blessed face,
Life is not life. Remember, Lord, and see,
Look back, embrace.

Nevertheless a cloud cut off their gaze;
They tarry to build up Jerusalem,
Watching for Him, while thro' appointed days
He watches them.

They do His will, and doing it, rejoice,
Patiently glad to spend, and to be spent;
Still He speaks to them, still they hear His voice,
And are content. —*C. Rossetti.*

### Notes and Queries.

The Editor is always willing to answer in this column, without any fee, all reasonable questions, as soon as possible.

But the following rules must be carefully observed:—

1. Every communication must be accompanied by the name and address of the writer.
2. The question must always bear upon nursing, directly or indirectly.

If an answer is required by letter a fee of half-a-crown must be enclosed with the note containing the inquiry.

*Solutions.*

(74) Would you kindly inform me: 1. How to make 1-1,000 perchloride into 1-5,000? 2. Does it mean 1 oz. to a pint of water? 3. What are quinine powders given for besides as a tonic?—*F. D.*

1. Add four times the original quantity of water to the solution. 2. It means 1 oz. in 5,000 oz., or 250 pints. 3. Quinine is best known as the remedy for and against malarial fevers, but it is also used for various other things.

*Loans.*

(75) Will you kindly tell me if there is a society which will grant a loan of money to hospital nurses? Some time ago I read in The Hospital that nurses, on becoming members of a society, could obtain money, to be returned with interest. I should be so much obliged if you can tell me of it.—*L. J. V.*

Do you mean the Royal National Pension Fund for Nurses? If so, the address is 28, Finsbury Pavement, E.C. Loans are made to members of this society by the Junius Morgan Benevolent Fund for certain approved purposes—that is, if the applicant is eligible and in distress.

*Health Lecturer.*

(76) Would you kindly inform me what are the necessary subjects and training required by a qualified lecturer on health subjects? I could not leave my work to go to London for any training. I would be glad to hear if any London society held examinations in this town from which I could get a certificate.—*Effleurage.*

You might write to the National Health Society, 53, Berners Street, W., for particulars as to their examinations and certificate.

*Premiums.*

(77) Can a nurse train for maternity work at Queen Charlotte's Hospital without paying a premium? 2. What is the difference between maternity and midwifery training?—*V. G.*

All pupils trained at Queen Charlotte's pay fees. 2. A maternity training fits the nurse to attend a case under a medical man; a midwife undertakes a simple confinement without medical supervision.

*L.O.S.*

(78) 1. Is a candidate eligible for examination for the L.O.S. certificate having only attended or seen 12 or 14 cases? If not, what number are required to be attended? 2. Has the superintendent nurse who instructs the candidate power to alter the number? 3. If not, and the candidate passes on a wrong statement of the number attended will it affect either her or the superintendent nurse in any way?—*F. L.*

A candidate for the L.O.S. examination must have attended not less than 20 labours. 2. Certainly not. 3. It will be a fraud.

*L.O.S.*

(79) Will you kindly tell me the address of the L.O. Society, and also state which is considered the highest degree in London for a midwife to take?—*Certificated Nurse.*

The L.O.S. is the one public body granting certificates to midwives. The large hospitals—like Queen Charlotte's—give certificates only to their own pupils. The address of the L.O.S. is 20, Hanover Square, W.

*Disinfecting Chamber.*

(80) Can you kindly tell me how a disinfecting chamber is to be worked, as it will be part of my duties here?—*Nurse P. O.*

There are many different machines. The makers supply full directions, but any disinfecting chamber worked by steam is sure to be under the management of the engineer. The principle applied to all, so far as the nurse is concerned, is that the articles, after being passed through the disinfector, must not be touched by nor brought in contact with anything soiled or infected.

*Lepers.*

(81) Can you inform me whether any nurses are required for the lepers? I am very anxious to go out to them, but cannot get any information about them. I have had some training, and should be so grateful if you could help me.—*Gladys M.*

Each leper hospital appoints its own nurses, when there are any, according to its own rules, and such rules can only be ascertained by application. But one thing is certain, you ought to be fully trained before endeavouring to nurse such a disease. "In a Leper Hospital" ("Mirror," January 22nd, 1898) gives a vivid impression of the nursing difficulties at one of the best managed settlements in Australia.

**Standard Books of Reference.**

"The Nursing Profession: How and Where to Train." 2s. net.
"The Nurses' Dictionary of Medical Terms." 2s.
"Burdett's Series of Nursing Text-Books." 1s. each.
"A Handbook for Nurses." (Illustrated.) 5s.
"Nursing: Its Theory and Practice." New Edition. 3s. 6d.
"Helps in Sickness and to Health." Fifteenth Thousand. 5s.

All these are published by The Scientific Press, Ltd., and may be obtained through any bookseller or direct from the publishers, 28 & 29, Southampton Street, London, W.C.

### Travel Notes.

### L.—IN THE LOIRE COUNTRY.

At Tours you may remain several days with advantage, for it is an admirable centre for excursions. It is only some twelve miles from Azay-le-Rideau, and about twenty-two from Chinon. On the first day devote yourself to the study of the historic town and castle of Loches.

### LOCHES, THE TOWN AND FORTRESS.

Loches is not more than twenty miles south of Tours, but I should advise your training one way, to be decided according to the convenience of hours, to make acquaintance with the curious double railway carriages, mounted one on the other, from which you obtain an extensive view of the surrounding country. After the smiling prosperity and peace of Azay-le-Rideau and Langeais, the frowning fortress of Loches seems to come almost as a shock. It is full of sinister memories; as the state prison of Louis XI. it shared with Plessis-les-Tours that wily monarch's favour, as a "safe bind, safe find" residence. Here Cardinal Balere was confined in a cage, which he had obligingly designed—at his master's request—for the occupation of others, and found too late that he had been contriving his own prison. The enormous stanchions on which it rested are still to be seen on the walls of a well-like dungeon in the centre of the castle. In this same castle Ludovico Sforza languished for 12 years, being released only to die. In his dungeon is a touching inscription on the walls, "Je ne suis pas content," but I think the characters are too modern to be those of Sforza, though the sentiment, alas! fits his condition only too well. Here, too, Philip de Commines, the gifted historian, was confined in 1486. Almost the only gentle memory connected with Loches is that of the frail, but loving, Agnes Sorrel, who, whatever her sins, did much to expiate them by her gentle influence in the fierce Court of Charles VII. The *oubliettes* of all shapes and designs and degrees of horror are to be found here in every part of the building, and an aged attendant told me that in his father's time bones were still found in unexpected places, generally in the two towers called La Tour Ronde and Le Marklet. The dungeons under Ludovico Sforza's prison are surpassingly dark and terrible, and as one goes down hundreds of feet into the very bowels of the earth one feels that all who thus descended to their doom in olden days must have tasted the bitterness of death, but for the most part the poor creatures made but one step from the top to the bottom, through convenient holes in the upper floors thoughtfully constructed for the purpose. It is well we have left these good old times behind, so often loudly lamented by us, from the midst of our nineteenth century comfort and safety.

### AGNES SORREL.

In a more modern building at the other end of the Castle platform in a tower is the very beautiful monument of Agnes Sorrel. She died in 1450, and the monks of Loches, who had long enjoyed her generous benefactions, desired in an access of hypocritical purity to remove her remains, but Louis XI., odious as he was, could not support such ingratitude, and told the monks that if they ejected her bones they must also give up the money and lands she had given them. Naturally, the grave was left intact up to the time of the Terror, when it shared the fate of so many others.

### THE CHURCH OF ST. OUEN AND THE TOWN.

St. Ouen is considered extremely interesting to architects from the singularity of its construction. It stands close to the Sous-Préfecture, and has four conical roofs of different heights. There is a fine Romanesque doorway, with unusually rich mouldings and sculptures, and a series of low arches in the west tower which lead into the nave. It is considered that the church was finished in 1180, but there are signs of much earlier times in the west tower and crypt. The gloomy fanatic, Louis XI., used this last frequently as a place of devotion when staying at Loches to gloat over the sufferings of his captives. The town is quaint and picturesque, and possesses a very good fortified gate. One is glad to emerge from the tragic memories of the castle into the wholesome sunshine again and spend an hour in the little town gazing into the somewhat shabby shops, and even then the effect of these massive walls and towers remains with us! they cling like some foul parasite to the rocks above the town in sinister power, and one feels as if an octopus tentacle might dart out, catch us in its grip and immure us for ever from the blessed light of day. Let us, therefore, return to Tours and to what the charming Howells calls the "cheerful overcharges" of the modern hotel.

### TOURS.

Tours is a gay little city, and full of convenient trams for the explorer, so that valuable time is not wasted in getting about. In the town itself your first point of observation will be the Cathedral, commenced in the twelfth century. The west front, of a much later date, and the glorious coloured glass, will repay study; observe closely the windows round the choir. The cloisters, of which guide books make nothing, are singular and very picturesque. In the Rue de Commerce stands the reputed house of Tristan l'Hermite, the executioner of Louis XI. As a devout pilgrim I hastened to see it at once, and though probably the foundations and walls are of that period, it has been much altered and added to, and has now the appearance of sixteenth century work. He probably lived somewhere close by, if not actually on that spot, for it is handy for Plessis-les-Tours, where he was frequently professionally engaged. That celebrated castle, so familiar to all lovers of "Quentin Durward," is about two miles from Tours, the road to it leading through delightful orchards. Little is now left of the vast fortifications of that place of evil repute; the moats, escarpments, ditches, and mantraps which once guarded this Royal dwelling, in accordance with the guilty fears of Louis, have entirely disappeared; but all his precautions could not avert death, and here, in 1483, that dark and wily spirit breathed his last. In the top storey of the chateau one sees the guard-room of the Scottish archers, and seem to hear again the bragging tones of Le Balafie and the sly *double entendre* of Maitre Pierre. Still higher, reached by a spiral staircase, is the tower where Charles VIII. spent much of his neglected youth. In the garden surrounding this Royal Den of Plessis-les-Tours there are vestiges only of a beautiful cloister, and, as usual, some gruesome dungeons; most of the trees have disappeared, and those that remain do not bear the unpleasant fruit which so upset the stout Scottish stomach of Quentin Durward.

### TRAVEL NOTES AND QUERIES.

RULES IN REGARD TO CORRESPONDENCE FOR THIS SECTION.—All questioners must use a pseudonym for publication, but the communication must also bear the writer's own name and address as well, which will be regarded as confidential. All such communications to be addressed "Travel Editor, 'Nursing Mirror,' 28, Southampton Street, Strand." No charge will be made for inserting and answering questions in the inquiry column, and all will be answered in rotation as space permits. If an answer by letter is required, a stamped and addressed envelope must be enclosed, together with 2s. 6d., which fee will be devoted to the objects of the "Hospital Convalescent Fund." Any inquiries reaching the office after Monday cannot be answered in "The Mirror" of the current week.

ITALIAN LAKES (Spera).—May is the most agreeable month undoubtedly, but failing that September and October are second best. It is difficult to say which is the prettiest, as it is purely a matter of taste. Como is on the whole perhaps the favourite, but Maggiore is superb, and the Lago di Gardu is a gem. If as you propose you go to Switzerland for the hot months, you could drop down to the lakes in the first week of September.

NAUHEIM, JOURNEY AND ACCOMMODATION (Nauheim).—Viâ Harwich, the Hook, and Frankfort, first-class, £3 8s. 3d.; second-class, £2 7s. 2d.; first, return (30 days), £4 18s. 5d.; second, return, £3 7s. 11d. You can certainly stay at an hotel, and they are not expensive. I do not think there are any pensions, but a few private apartments, Write for prices to Hotel Goldener Engel and the Curhaus and Bellevue. I do not think there is any hydropathic establishment. There is a visitor's tax of 12 marks for one person and 18 for two, with an extra 3 marks for every additional member of a party. It is a small place, but bright and gay, and not so expensive as Carlsbad, &c. English Church service in the summer, and a library.

## Figures and Tables

**Fig. 12. f1:**
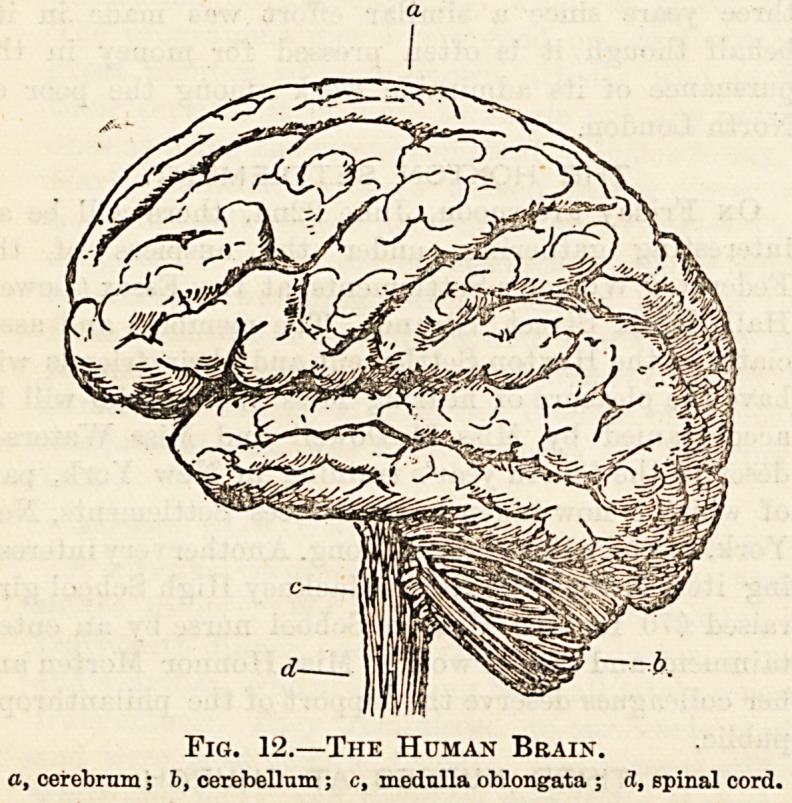


**Fig. 13. f2:**